# A high performance liquid chromatographic assay of Mefloquine in saliva after a single oral dose in healthy adult Africans

**DOI:** 10.1186/1475-2875-11-59

**Published:** 2012-02-27

**Authors:** Grace O Gbotosho, Christian T Happi, Omowunmi Lawal, Abayomi Sijuade, Akin Sowunmi, Ayoade Oduola

**Affiliations:** 1 Department of Pharmacology and Therapeutics, College of Medicine, University of Ibadan, Ibadan, Nigeria; 2 Malaria Research Laboratories, Institute for Advanced Medical Research and Training, College of Medicine, University of Ibadan, Ibadan, Nigeria; 3 Stewardship for Research, Tropical Disease Research (TDR), World Health Organization, Geneva, Switzerland

**Keywords:** Malaria, Mefloquine, Saliva, High performance liquid chromatography

## Abstract

**Background:**

Mefloquine-artesunate is a formulation of artemisinin based combination therapy (ACT) recommended by the World Health Organization and historically the first ACT used clinically. The use of ACT demands constant monitoring of therapeutic efficacies and drug levels, in order to ensure that optimum drug exposure is achieved and detect reduced susceptibility to these drugs. Quantification of anti-malarial drugs in biological fluids other than blood would provide a more readily applicable method of therapeutic drug monitoring in developing endemic countries. Efforts in this study were devoted to the development of a simple, field applicable, non-invasive method for assay of mefloquine in saliva.

**Methods:**

A high performance liquid chromatographic method with UV detection at 220 nm for assaying mefloquine in saliva was developed and validated by comparing mefloquine concentrations in saliva and plasma samples from four healthy volunteers who received single oral dose of mefloquine. Verapamil was used as internal standard. Chromatographic separation was achieved using a Hypersil ODS column.

**Results:**

Extraction recoveries of mefloquine in plasma or saliva were 76-86% or 83-93% respectively. Limit of quantification of mefloquine was 20 ng/ml. Agreement between salivary and plasma mefloquine concentrations was satisfactory (r = 0.88, *p *< 0.001). Saliva:plasma concentrations ratio was 0.42.

**Conclusion:**

Disposition of mefloquine in saliva paralleled that in plasma, making salivary quantification of mefloquine potentially useful in therapeutic drug monitoring.

## Background

The morbidity and mortality associated with malaria is highest in African children with approximately one million deaths per year. The spread of drug resistant *Plasmodium falciparum *confounds malaria control efforts and has resulted in the widespread use of artemisinin based combination therapy (ACT) in the management of malaria in several sub-Saharan African countries [1]. Mefloquine-artesunate is a formulation of ACT recommended by the World Health Organization [1] and historically the first ACT used clinically. The combination remains an effective treatment for uncomplicated malaria [2-5]. The effectiveness of ACT is dependent on the different modes of action of the drugs in the combination. The artemisinin derivatives cause an initial rapid reduction in parasite biomass though they are rapidly eliminated while the partner drugs such as mefloquine, lumefantrine, piperaquine, or amodiaquine are more slowly eliminated and cause subsequent removal of the remaining parasites. Thus, when combinations of drugs with discordant half-lives are used, the patient in effect receives monotherapy with the longer lasting drug for the tail end of therapy [4]. The overall cure rates, therefore, depend upon there being sufficient partner drug to remove the residual parasite biomass left by the artemisinin derivative. Thus, it is important that the non-artemisinin partner drug maintains consistent pharmacokinetic and pharmacodynamic properties.

Based on this backdrop, the use of ACT demands constant monitoring of therapeutic efficacies and drug levels, in order to ensure that optimum drug exposure is achieved with the dosing strategy and detect reduced susceptibility to these drugs. Traditionally, anti-malarial drug levels have been measured in biological fluids such as whole blood, plasma or red blood cells [2,6-11] obtained by invasive techniques. This method of sampling poses challenges in resource poor settings and during epidemiological studies where repeated measurements are required. Thus, quantification of anti-malarial drugs in biological fluids other than blood would provide a more readily applicable method of therapeutic drug monitoring in developing endemic countries. The use of saliva as an alternative biological fluid in therapeutic drug monitoring is justified by the simplicity in obtaining samples from patients in a non-invasive manner and the possibility of home monitoring. Whole saliva can be collected by individuals with limited training and no special equipment is required to collect the fluid, thus saliva represents a potentially useful matrix for estimating drug levels [12,13]. The major mechanism by which a drug appears in saliva is thought to be through passive diffusion across a concentration gradient and only the unbound fraction of the drug in serum is available for diffusion into saliva [14]. The unbound fraction of a drug is usually the pharmacologically active fraction and this may represent an advantage of drug monitoring in saliva in comparison with drug monitoring in serum where both bound and unbound drug can be detected [15].

Methods to measure mefloquine in whole capillary blood and plasma have been previously described [16-18], however, these are fraught with limitations. For instance, sample collection is by invasive techniques in most cases, some methods require large sample volume, while others require solid phase extraction. There is no documented report on estimation of mefloquine concentrations in saliva. Thus in this study, efforts were devoted to the development of a simple, field applicable, non-invasive method for assay of mefloquine in saliva and its potential use for therapeutic drug monitoring in resource poor settings.

## Methods

### Chemicals

Mefloquine and verapamil (internal standard) were obtained from Walter Reed Army Research Institute, USA and their purity was ≥ 99%. All solvents used were HPLC grade (from Sigma-Aldrich, Milwaukee, USA), while phosphoric acid and all other reagents employed were analytical grade (BDH, Polle, UK). Mefloquine tablets (Mepha Limited, Aesch-Basel, Switzerland) were purchased locally from a wholesale Pharmacy in Ibadan, Nigeria.

### Instrumentation

The separation of mefloquine was carried out under isocratic condition at room temperature. The HPLC system consisted of a Cecil 4100 pump, attached to Cecil 4200 variable wavelength UV-Visible detector set at 220 nm. The chromatograms were recorded and analysed with PowerStream software (CE 4900) provided with the instrument (Cecil Instrument, Cambridge, UK). Chromatographic separation was achieved using a Hypersil ODS column (4.6 mm × 250 mm; particle size 5 μm). The mobile phase consisted of phosphate buffer-acetonitrile-methanol (40:30:30 v/v/v) with 1% triethylamine adjusted to a pH 2.8 with concentrated phosphoric acid. The flow rate of mobile phase was 1.0 ml/min.

### Calibration and sample preparation

Stock solutions (1 mg/ml) of mefloquine and verapamil were prepared in 70% methanol and stored at 4°C. Working solutions of different concentrations were prepared from the stock. Drug free human plasma or whole unstimulated saliva (400 μl) was spiked with the working solution of mefloquine to yield final concentrations of 3,000, 2,000, 1,000, 800, 600, 400, 200 and 0 ng/ml of mefloquine. The resulting solutions were used to develop and evaluate the method. The pH of saliva samples was measured using a pH meter (Mettler, Switzerland).

### Extraction procedure

To plasma or saliva (0.4 ml) in a screw capped 15 ml polypropylene centrifuge tube, 10 μl (1000 ng) of 100 μg/ml verapamil was added as internal standard. The mixture was vortexed for 30 seconds. Two milliliters (2 ml) of acetonitrile was added to the mixture followed by 0.5 ml glycine buffer (pH 9.2). The mixture was vortexed and centrifuged for 5 minutes. Supernatant was thereafter removed and transferred into clean tubes. Two milliliters (2 ml) of dichloromethane was added and the mixture vortexed for 2 mins and thereafter centrifuged. After centrifugation (1,000 g × 10 min) and separation, the organic phase was evaporated to dryness at 37°C under a steady stream of nitrogen. The residue was reconstituted in 75 μl mobile phase and 40 μl was injected onto the column.

### Precision, accuracy and recovery

Sample preparation and extraction were performed on four replicates at each concentration of mefloquine on each of four days. Calibration curves were prepared from the measurement of peak height ratios of the analytes and internal standard. To assess precision and reproducibility of the method, coefficients of variation (CVs) and standard deviations were determined for intra- and inter-assay variability. Recovery was determined for each concentration by comparison of peak height ratio of the extracted known standards with the directly injected standard concentrations.

### Interference

Commonly used anti-malarial drugs including chloroquine, pyrimethamine, quinine, amodiaquine, sulphadoxine were studied for interference by spiking the drugs in blank plasma or saliva. The drugs were extracted according to the method described above. The presence of peaks was monitored after injection of 40 μl of the reconstituted sample.

### Clinical evaluation

The study protocol was approved by the Joint Ethical Review Committee of the University of Ibadan/University College Hospital, Ibadan and written informed consent was obtained from each subject prior to inclusion. All subjects underwent a routine clinical examination prior to inclusion. Four apparently healthy adult subjects were enrolled into the study. Subjects had no history of drug ingestion in the preceding 28 days and each subject fasted overnight. The demographic data of subjects such as temperature, weight and age were recorded before drug administration. Each subject received standard single oral dose of 20 mg/kg mefloquine base. Venous blood samples (5 ml) and 1 ml whole unstimulated saliva were obtained from each of the four volunteers. Venous blood samples were collected into heparinised tubes at 0 hr before drug administration, then at 0.5, 1.0, 2.0, 3.0, 4.0, 6.0, 8.0, 10.0, 12.0, 36.0, 48.0, 72.0, 168, 336, and 504 hr after drug administration. Saliva samples were collected simultaneously at the same times as venous blood samples. All subjects rinsed their mouth with water before producing saliva. The pH of saliva was determined throughout the sampling period. Blood was centrifuged (2000 g for 20 min) and the plasma was removed and stored at -20°C until analysed. Samples were extracted as described above.

## Results

### Chromatographs

**Figure 1:**
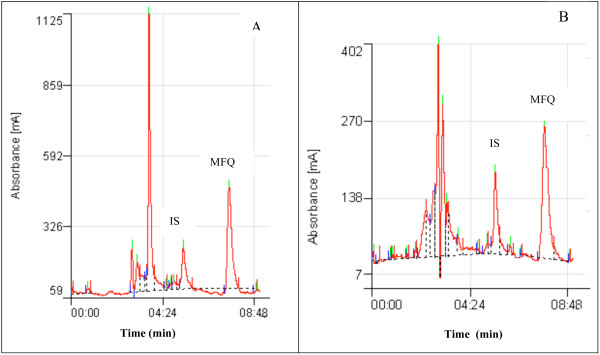
**Separation of mefloquine (MFQ) and internal standard (IS) from drug free plasma (A) and saliva (B) spiked with 100 μg/ml of mefloquine**.

**Figure 2:**
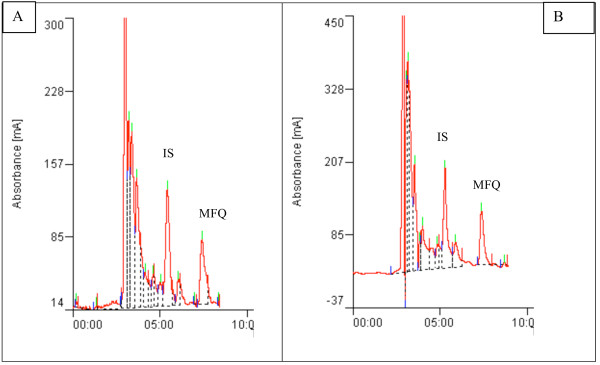
**Chromatogram of mefloquine (MFQ) and internal standard (IS) in plasma (A) and saliva (B) samples obtained from a volunteer 6 hrs after a single oral dose of mefloquine (20 mg/kg body weight)**.

The calibration curves for mefloquine spiked in drug free plasma or saliva exhibited good linearity. Linear regression analysis yielded correlation coefficients r^2 ^> 0.98 in plasma and r^2 ^> 0.96 in saliva. The baseline of mefloquine and the internal standard were well resolved at the calibration ranges of 0 to 3000 ng/ml with retention times of 5.0 and 7.0 min for verapamil and mefloquine respectively. The separation chromatograms of mefloquine and the internal standard from spiked plasma or saliva samples (Figure 1) corresponded with those of plasma or saliva samples obtained from a healthy volunteer 6 hr after an oral dose of mefloquine (Figure 2). All peaks were baseline resolved and clear of interference from endogenous component of samples.

### Recovery, calibration curves and reproducibility

The percentage recovery of mefloquine ranged from 76-86% and 83-93% in plasma and saliva respectively. The extraction recoveries for 400 ng/ml, 800 ng/ml and 1,000 ng/ml of mefloquine in plasma or saliva were 75.7 ± 6.2% vs 83.3 ± 5.6%, 79.2 ± 4.2% vs 91.3 ± 1.7% and 86.2 ± 2.0% vs 92.8 ± 2.0% (n = 4) respectively. The intra and inter assay variation of spiked plasma samples were 1.68% (n = 4) and 7.40% (n = 4) respectively at 200 ng/ml and 4.76% (n = 4) and 2.64% (n = 4) respectively at 1000 ng/ml. The intra- and inter-assay variation of spiked saliva samples were 1.72% (n = 5) and 4.02% (n = 5) respectively at 200 ng/ml and 7.00% (n = 4) and 6.62% (n = 4) respectively at 1000 ng/ml. The limit of quantification of the assay was 20 ng/ml. The limit of detection of the assay was 6.25 ng/ml as measured by a peak corresponding to four times the size of baseline noise at 0.01 absorbance units full-scale (aufs).

### Interference

There was no interference from endogenous compounds or any of the commonly used anti-malarial, analgesic and anti-infective drugs with the peaks of mefloquine or the internal standard.

### Stability

Comparative short term stability tests were performed to simulate the effect of different storage and processing conditions on the stability of mefloquine in saliva versus plasma. Samples were stored at 37°C, 45°C, room temperature and also made to undergo three freeze-thaw cycles. The concentrations of mefloquine in these samples were not affected by different storage or processing conditions.

### Clinical application

Four apparently healthy male subjects were recruited for this aspect of the study. The mean age and weight of subjects were 29 years ± 0.4 and 64.5 kg ± 9.9 respectively. Each subject received 20 mg/kg body weight mefloquine and this was approximately 1,100 mg to 1,480 mg mefloquine base.

### The pH of saliva samples collected from healthy subjects

The pH of saliva in the four healthy subjects measured during the study ranged between 6.4-8.0. The mean pH of saliva was 7.8 ± 0.1, 7.7 ± 0.3, 7.1 ± 0.3 and 6.8 ± 0.3 respectively in each of the four subjects. There was a slightly higher value of the pH of saliva in two subjects compared with the normal pH of saliva which ranges between 6.2-7.4.

### Pharmacokinetic disposition of Mefloquine in plasma and saliva

**Figure 3:**
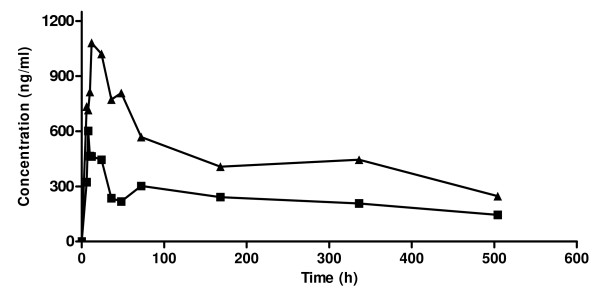
**Concentration - time curve for mefloquine in plasma (▲) and saliva (■) after administration of single oral dose of mefloquine (20 mg/kg body weight) in healthy volunteers**.

The pharmacokinetic disposition of mefloquine in plasma and saliva of healthy volunteers is described in Table 1. There was inter-individual variability in mefloquine disposition. Mefloquine was detectable in plasma and saliva within 30 minutes of oral administration and reached a maximum concentration within 6-12 hours of drug administration. The mean time to reach a maximum concentration in plasma and saliva were similar (10.0 ± 1.4 hr vs 10.0 ± 1.1 hr, *p *= 0.999). The mean maximum concentration of mefloquine in plasma was almost twice the concentration in saliva (1,016.8 ± 151 ng/ml vs 571.5 ± 77.3 ng/ml, *p *= 0.066) although the difference was not statistically significant probably due to sample size. Following attainment of the peak concentration, a decline in mefloquine concentration was observed and the decline phase of the saliva concentration time curve were approximately parallel to that in plasma (Figure 3). The mean saliva concentration of mefloquine was approximately 2/5 that in plasma.

**Table 1:** Comparative pharmacokinetic parameters of mefloquine in plasma and saliva of healthy adult volunteers after a single oral dose of mefloquine

Parameters	Plasma n = 4	Saliva n = 4	p value
**Tmax **(h)	10.0 ± 1.4	10.0 ± 1.1	1.000
Range	6.0-12.0	8.0-12.0	

**Cmax **(ng/ml)	1016.8 ± 151.04	571.5 ± 77.0	0.066
Range	675.8-1367.2	437.5-705.4	

**t_1/2 _**(h)	274.3 ± 40.4.4	248.8 ± 39.4	0.678
Range	175.2-353.1	202.3-327.2	

**AUC **(ng/ml.h)	204191.6 ± 11629.6	104253.0 ± 7848.1	0.001
Range	185448.9-237144.0	95562.3-119918.0	

**CL **(ml/h/kg)	87.2 ± 16.8	171.7 ± 7.3	0.010
Range	53.7-133.9	157.1-181.3	

The mean half-life of mefloquine in plasma and, saliva were statistically indistinguishable (274 ± 40.4 hr vs 248.8 ± 39.4 hr, *p *= 0.678). However, there was a statistically significant difference between the mean AUC for mefloquine in saliva and, plasma. The plasma AUC was approximately twice the saliva AUC (204191.6 ± 11629.6 ng/ml.h vs 104253.0 ± 7484.1 ng/ml.h, *p *= 0.001). The oral clearance of mefloquine was significantly higher in saliva compared with plasma (*p *= 0.010). The relationship between instantaneous concentration of mefloquine in saliva and plasma was assessed by regression analysis and the correlation coefficient was 0.88 (*p *= 0.0002). The mean saliva/plasma ratio for all subjects at all time points was 0.42 ± 0.17. Our studies did not reveal a concentration or time dependent saliva/plasma ratio (r = 0.2480, *p *= 0.463 and r = 0.440, *p *= 0.176 respectively).

## Discussion

Therapeutic drug monitoring in saliva offers a painless, non-invasive and cost effective means of estimating the free drug concentration in plasma or serum. Unfortunately this biological matrix is infrequently used despite its potential usefulness. In recent times however, there has been increased interest in the use of this alternative matrix for drug treatment testing [12,13,19]. The need for the use of oral fluid in therapeutic monitoring of anti-malarial drugs in resource poor malaria endemic regions cannot be overemphasized. Although, limited studies have described the pharmacokinetic disposition of anti-malarial drugs, such as quinine and chloroquine, in saliva [20,21], this biological matrix is seldom used for therapeutic drug monitoring. More recently amodiaquine disposition in saliva was also reported [22], but no study has described mefloquine determination in saliva.

The assay described in this paper is a simple and cost effective method for the analysis of mefloquine in saliva and this is the first report of mefloquine determination in saliva. A two-step extraction procedure was developed and it was sufficiently sensitive to detect both mefloquine and the internal standard used (verapamil) at a wavelength of 220 nm. The extraction solvents gave optimal recovery of mefloquine and internal standard from saliva and plasma samples. The advantages of this assay are that it is relatively inexpensive, sample collection and processing is rapid and simple and the technique applicable in field settings in resource-poor countries. Sensitivity and selectivity are also retained which is necessary for accurate determination of mefloquine in saliva.

It is noteworthy that mefloquine was measurable in saliva within 30 minutes of oral administration such as has been reported for quinine and amodiaquine [20,22], and it was detectable for up to 504 hours after a single oral dose of 20 mg/kg mefloquine. The concentration of mefloquine in saliva which represents the free or unbound fraction was approximately two-fifth that of plasma. This would suggest that in these healthy volunteers mefloquine was probably 60% protein bound. This value is less than the reported extent of protein binding of mefloquine (> 98%) [23] and variations in salivary pH may have accounted for this discrepancy. Passive diffusion across a concentration gradient is thought to be the major mechanism accounting for the appearance of a drug in saliva. The pKa of a drug and the pH gradient between plasma and saliva determine the concentration gradient on both sides of the cell membrane and influence the availability of a drug in saliva [24]. The pH of saliva measured during the study was in the range of 6.4 to 8.0 (mean = 7.3 ± 0.25). The presence of a drug in saliva is influenced by various factors, which include the physicochemical properties of the drug, lipid solubility, salivary pH and the degree of protein binding of drug [25]. These factors may have limited the widespread use of salivary drug concentrations for therapeutic drug monitoring. In two of the volunteers, salivary pH range (7.5-8.0, mean = 7.8 ± 0.1 and 7.0-8.0, mean = 7.7 ± 0.3) was higher than normal saliva pH (6.0-7.4).

Equilibration between saliva and plasma concentrations was rapid and the decline phase of mefloquine in saliva concentration time-curve was approximately parallel to that in plasma in this study. The C_max _and the AUC_0-504 h _values obtained from the saliva data were about half those obtained from plasma while the T_max _of mefloquine determined from both fluids were similar and these results were consistent with previous studies on pharmacokinetics of chloroquine in saliva [21]. The saliva clearance rate of mefloquine was about twice the plasma clearance rate such as has also been reported for chloroquine [21]. The salivary concentration-time profile reflects the plasma concentration-time profile. The apparent first order elimination rate constant from salivary concentrations derived from the regression of ln (salivary concentration) on time was 0.0029 ± 0.0007 h^-1 ^while the corresponding value derived from plasma was statistically indistinguishable (0.0027 ± 0.0009 h^-1^, *p *= 0.776). Thus it is possible to derive the apparent first order elimination rate constant from the salivary measurements alone. The applicability of the method in studying pharmacokinetic disposition of mefloquine in saliva of volunteers suggests that the method may be suitable in developing disease endemic areas.

A fundamental prerequisite for the application of saliva in therapeutic drug monitoring is a definable relationship between the concentration of a therapeutic drug in blood (serum) and the concentration in saliva. This was established in the present study where a significant correlation (r = 0.88) was shown to exist between salivary and plasma mefloquine levels. Similarly, previous reports of salivary quantification of artemisinin [26] have shown that concentration of artemisinin in saliva was comparable to its unbound concentration in plasma (r = 0.85). The application of saliva for monitoring drug levels has been the subject of considerable investigation. Several studies have described the usefulness of saliva in monitoring patient compliance and drug levels of psychiatric medications, antiepileptic drugs, anticancer drugs, and also for the evaluation of illicit drug use [27-29].

## Conclusion

The assay described in this paper is a simple and sensitive method for analysis of mefloquine in saliva, which appears to enter the saliva passively. The disposition of mefloquine in saliva appears to be similar to its disposition in plasma thus measurement of mefloquine in saliva provides a valuable option in therapeutic drug monitoring. Further pharmacokinetic studies are however required to evaluate mefloquine concentrations in saliva during malaria infection in a large population. In addition, evaluation of the potential to simultaneously quantify artesunate in saliva samples during combination therapy with mefloquine is important.

## Competing interests

The authors declare that they have no competing interests.

## Authors' contributions

GOG was responsible for developing research protocol, data analysis, and manuscript preparation, CH participated in developing research protocol, data analysis and manuscript preparation. OL participated in data collection and data analysis, AOS participated in data collection, data analysis and manuscript preparation. AS was responsible for conceptualization, design of the study and participated in manuscript preparation. AO participated in development of research protocol. All authors read and approved the final manuscript.
